# Electrochemical Detection of Arsenite Using a Silica Nanoparticles-Modified Screen-Printed Carbon Electrode

**DOI:** 10.3390/ma13143168

**Published:** 2020-07-16

**Authors:** Suhainie Ismail, Nor Azah Yusof, Jaafar Abdullah, Siti Fatimah Abd Rahman

**Affiliations:** 1 Institute of Advanced Technology, Universiti Putra Malaysia, Selangor, Serdang 43400, Malaysia; suhainie90@gmail.com (S.I.); jafar@upm.edu.my (J.A.); 2 Department of Chemistry, Faculty of Science, Universiti Putra Malaysia, Selangor, Serdang 43400, Malaysia

**Keywords:** arsenite, electrochemical sensor, screen-printed carbon electrode, silica nanoparticles

## Abstract

Arsenic poisoning in the environment can cause severe effects on human health, hence detection is crucial. An electrochemical-based portable assessment of arsenic contamination is the ability to identify arsenite (As(III)). To achieve this, a low-cost electroanalytical assay for the detection of As(III) utilizing a silica nanoparticles (SiNPs)-modified screen-printed carbon electrode (SPCE) was developed. The morphological and elemental analysis of functionalized SiNPs and a SiNPs/SPCE-modified sensor was studied using field emission scanning electron microscopy (FESEM), transmission electron microscopy (TEM), energy dispersive X-ray spectroscopy (EDX), and Fourier transform infrared spectroscopy (FTIR). The electrochemical responses towards arsenic detection were measured using the cyclic voltammetry (CV) and linear sweep anodic stripping voltammetry (LSASV) techniques. Under optimized conditions, the anodic peak current was proportional to the As(III) concentration over a wide linear range of 5 to 30 µg/L, with a detection limit of 6.2 µg/L. The suggested approach was effectively valid for the testing of As(III) found within the real water samples with good reproducibility and stability.

## 1. Introduction

Arsenic, a highly toxic element, is commonly found in many different minerals. Most arsenic compounds are often detected as a by-product of copper and lead refining. In the electronics industry, small quantities of arsenic can be mixed with germanium and silicon by heating, resulting in a sublime arsenic and the end product of iron (II) sulfide for transistor production. Small amounts of arsenic are essential to the electronics industry—heating a mixture of arsenic, germanium, and silicon for the purpose of transistor production causes arsenic to sublime and leave behind iron (II) sulfide. On the other hand, the arsenic-gallium arsenide (GaAs) compound is used to make light-emitting diodes (LEDs). LEDs produce the lit numbers in hand-held calculators, clocks, watches, and other electronic devices. Despite its benefits, arsenic pollution can endanger human lives and affect the sustainability of the ecosystem [1,2]. In fact, arsenic is four times more poisonous in comparison with mercury, a known harmful metal. Arsenic is mainly found in drinking water and in groundwater, and its contamination has affected more than 140 million people across 70 different countries [3]. Arsenic exists in four oxidation states: −III, 0, III, and V, and can be found in nature as organic or inorganic arsenic. Inorganic arsenic, namely As(V) and As(III), is categorized as the most dominant form of arsenic contamination in groundwater. Among both those forms, up to 80% toxicity of total arsenic is contributed by As(III) [4,5]. The toxicity of As(III) is 50 times higher than As(V) due to the enzymatic reaction in the human respiratory system [6]. Meanwhile, organic arsenic can be defined as arsenic atoms bonded with carbon elements. Organic arsenic is found mainly in seafood such as fish and shellfish, and is not harmful to human health [7].

Presently, numerous detection techniques have been established to detect and quantify As(III), such as atomic absorption spectrometry (AAS) [8], atomic fluorescence spectrometry (AFS) [9], and inductively coupled plasma mass spectrometry (ICP-MS) [10]. However, these techniques are unable to detect individual arsenic species and must be used with an upstream separation scheme. These techniques also possess limitations due to the high operational and maintenances costs, long analysis time, low selectivity and sensitivity, and can only be handled by a trained operator [11]. Thus, this highlights the necessity to develop a simple, economical, fast, selective, and precise detection method for trace levels of heavy metals [12]. Electrode-based electrochemical sensing systems satisfy many requirements for both qualitative and quantitative analyses to replace the conventional methods, as they offer the advantages of simple instrumentation, easy handling, rapid analysis, and portability. The use of screen-printed electrodes (SPEs) is found to be prominent for on-site detection, since the screen-printed technology enables mass production of highly reproducible, single-shot (disposable) electrodes [13,14]. Moreover, an easily modifiable SPEs configuration, including microelectrodes and chemically modified electrodes [15], allows the development of specific sensors that are suitable for decentralized assays. As such, SPEs are favored to be used as sensing surfaces due to their cost effectiveness, design versatility, and commercial availability in comparison with other types of electrodes [15,16]. Hence, the development of an inexpensive and reliable technology for the rapid determination of arsenic is crucial for monitoring the environment and human safety.

The design and construction of a nanoparticle-modified electrode could be the key factor to improving the sensitivity and selectivity of the electroanalytical measurement [17]. In this context, the modification of a sensing electrode using various materials, such as gold nanoparticles (AuNPs) [18], gold-palladium nanoparticles (Au-PdNPs) [19], gold-platinum nanoparticles (Au-PtNPs) [5], and silver nanoparticles (AgNPs) [20,21], was proven to be effective for arsenic quantification. Most of these approaches involve the use of metal-based nanoparticles for electrode modification, particularly for gold nanoparticles, since they provide a more sensitive anodic current reaction [17]. Considering that, a previous work had demonstrated the development of an electrochemical sensor utilizing gold nanoparticles combined with silica nanoparticles (Au-SiNPs) as a sensing platform for arsenic detection. In this two-steps of nanoparticles-modified screen-printed carbon electrode (SPCE) approach, the developed sensor showed good sensitivity and selectivity towards As(III) determination, with a low detection limit of 5.6 ppb [22]. Although the AuNPs-modified electrode has been widely used in arsenic determination, such electrodes still come with disadvantages, including the high cost of noble metals that makes the system inefficient for routine analysis, as well as the non-uniform coatings caused by the easy agglomeration of small-sized nanoparticles, therefore, complicating the fabrication protocols [23]. Since an SPCE-based disposable electrochemical assay may be of particular interest for the in-field detection of arsenic in a simple and cost-effective way, non-metals become a great alternative to replace metal-based nanomaterials for electrode modification. Moreover, non-metal-based nanomaterials have remarkable advantages, such as higher catalytic activities, good stability, and abundance in occurrence, all of which contribute to economical fabrication and their capability to act as a sensor [23,24].

**Figure 1:**
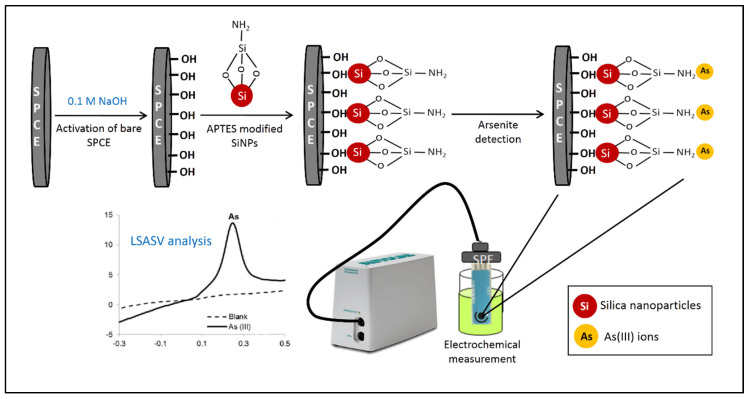
Schematic representation of the fabrication process of the silica nanoparticles (SiNPs)/screen-printed carbon electrode (SPCE) electrochemical sensor.

For the aforementioned reasons, the silica nanoparticles-based SPCE is seen to have great potential for use in the electrochemical detection of heavy metal ions, including As(III), in the environmental field. It is imperative to note that silica nanoparticles (SiNPs) have been established as one of the most promising support elements due to their high surface area, biocompatibility, and ease of functionalization that can further enhance the sensor performance [25]. Therefore, this work demonstrates the fabrication of SiNPs/SPCE-modified electrodes to narrow the gap of the unexplored strategy in improving the electrochemical detection of As(III) ions via the linear sweep anodic stripping voltammetry (LSASV) technique. The SPCE was modified with 3-aminopropyltriethoxysilane (APTES)-functionalized SiNPs through the drop-casting method, and subsequently applied as an electrochemical interface, as illustrated in Figure 1. Through this simple work-up process, the high specificity and sensitivity of the sensor can be achieved from the abundant amino (NH_2_)-containing active groups on the electrode surface, which are favorable for the LSASV determination of As(III) [25]. The proposed scheme can be introduced as a new route for a simple, fast, and practical method in the advancement of sensor studies from the existing scheme, in which the latter requires complicated fabrication protocols using high-cost materials.

Evidently, although the SiNPs/SPCE was unable to demonstrate the lowest limit of detection (LOD) in comparison with the previous reported works [5,22], the SiNPs/SPCE reported in this research had not only offered a competitive detection limit but a simplification of the analytical procedure through the alleviation of the requirement for electrode modification. Moreover, the proposed sensor had performed satisfactorily in a range of real samples analyses. It is worth noting that the combination of an SPCE with SiNPs, where the latter are low-cost materials with adequate efficiency and selectivity, may represent an interesting platform for an affordable and disposable detection system that can be extensively used for in-field applications of arsenic contamination monitoring.

## 2. Materials and Methods

### 2.1. Materials

All reagents were analytical grade and used without further purification. Ultrapure water (mili-Q ultrapure water system, Waltham, MA, USA) was used in all of the experiments. Tetraethylorthosilicate (Si(OC_2_H_5_)_4_) (98%) and 3-aminopropyltriethoxysilane (C_9_H_23_NO_3_Si) (99%) were obtained from Sigma Aldrich (St. Louis, MO, USA). Hydrochloride acid (HCl) (37%), ammonium hydroxide (NH_4_OH) (25%), sulphuric acid (H_2_SO_4_) (98%), and phosphate buffer (PBS) were purchased from R&M Chemicals (Essex, UK). Ethanol, (CH_3_O_4_) (95%) and As(III) standard solution (1000 mg/L) were obtained from HmbG Chemicals and Merck (Hamburg, Germany), respectively. The screen-printed carbon electrode (SPCE, DRP-C110DIEL) with a diameter of 4 mm and working area of 0.11 cm^2^ was purchased from Dropsense Company (Oviedo, Spain). The reference electrode was made of silver, while the working and auxiliary electrodes were made of carbon. All the electrodes were fabricated on a ceramic substrate (3.4 cm length × 1.0 cm width × 0.05 cm thick).

### 2.2. Instruments

Electrochemical studies were performed using a potentiostat electrochemical system (Eco Chemie B. V., Utrecht, The Netherlands) equipped with a computer-controlled µAUTOLAB III. The electrochemical data were analyzed by the NOVA 1.11 software (Eco Chemie B. V., Utrecht, Netherlands). Field emission scanning electron microscopy (FESEM) and energy dispersive X-ray (EDX) were performed on a JSM 7600F electron microscope (JEOL, Ltd., Tokyo, Japan). Transmission electron microscopy (TEM) was conducted on a H-7100 electron microscope (Hitachi, Ltd., Tokyo, Japan). The Fourier transform infrared spectrum (FTIR) was recorded using a Perkin Elmer spectroscopy (Waltham, MA, USA). In addition, the inductively coupled plasma mass spectrometry (ICP-MS) was conducted to validate the performance of the fabricated SiNPs/SPCE towards As(III) detection. All measurements were conducted at room temperature.

### 2.3. Synthesis and Fabrication of SiNPs-Modified SPCE

SiNPs were synthesized by mixing 47 mL of EtOH, 3.3 mL of NH_4_OH, and 4 mL of TEOS (4.38 M) under stirring. The mixture was incubated for 24 h at room temperature. Afterwards, 0.3 mL of 98% APTES (4.11 M) was added to the mixture and incubated overnight at room temperature. The solution was centrifuged at 40,000 rpm for 2 h and dried in a drying oven at 70 °C for 30 min to obtain APTES-functionalized SiNPs. Next, the electrode was pre-treated to remove the physically adsorbed impurities on the electrode surface by applying the potential of 0.1 to 0.7 V using the cyclic voltammetry (CV) technique in 0.1 M NaOH for 30 cycles, at a scan rate of 0.1 V/s, and dried at room temperature [26]. Prior to use, 3 mg of the product was sonicated with 1 mL of deionized water for 60 min. After that, the working electrode was prepared by drop-casting 10 µL of the SiNPs solution onto the surface and dried overnight at room temperature. The modified electrode was then thoroughly rinsed with deionized water to remove the excess SiNPs. Finally, the modified electrode was characterized using CV in 5 mM [Fe(CN)_6_]^3−/4−^ containing 0.1 M KCl as a supporting electrolyte at the potential range of −0.5 to 0.6 V with the scan rate of 0.1 V/s. Figure 1 illustrates the fabrication process of the SiNPs/SPCE for arsenite detection.

## 3. Results and Discussion

### 3.1. Characterization of SiNPs and SiNPs/SPCE

**Figure 2:**
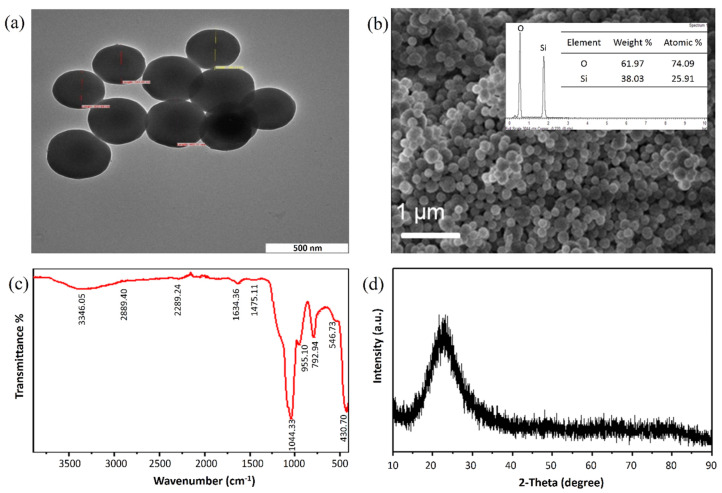
(**a**) TEM image, (**b**) FESEM image and the EDX profile (inset), (**c**) FTIR spectra, and (**d**) XRD pattern of the synthesized SiNPs.

The TEM image of the nanoparticles (Figure 2a) shows that the particles were monodispersed with sizes ranging from 200 to 250 nm and their size distribution was uniform with the spherical shape of the nanostructures. As reported by previous studies, the smaller size of SiNPs of less than 100 nm is well-known as an excellent modifier employed in constructing electrochemical sensors by providing high sensitivity and selectivity towards analyte detection [27]. Although the smaller size of SiNPs provides great advantages, the particles also possess some limitations due to the high surface energy and particle–particle interactions. The small particles show low colloidal stability and tend to aggregate very fast. This aggregation reduces the available surface area for the reduction of metal ions, As(III). In this work, although the synthesized SiNPs resulted in a larger size, the nanoparticles still exhibited extraordinary properties and offered unique features, as well as enhanced the performance of the developed sensor.

Meanwhile, in order to confirm the successful synthesizing of functionalized SiNPs, the surface morphology of SiNPs was examined using FESEM as shown in Figure 2b. The SiNPs particles were directly attached to a sample holder using a carbon paste coated with gold to reduce the charging effect. The SEM image showed the SiNPs were uniformly dispersed with spherical morphologies. The elemental composition of SiNPs was further obtained using EDX analysis as shown in Figure 2b (inset). Two strong peaks of O and Si were observed, which confirmed the presence of silica and oxygen with the weight percentages (wt %) of 38.03 and 61.97, respectively. This indicates that the synthesized silica has high purity [28].

The FTIR spectra of SiNPs were analyzed to study the functional group that exists in the synthesized SiNPs. As shown in Figure 2c, the absorption bands exhibited at 3346.65 and 1634.36 cm^−1^ were attributed to the stretching mode of NH_2_ and OH, respectively. Meanwhile, the characteristic band at 1044.33 cm^−1^ corresponded to the Si–O–Si symmetric stretching. The peaks at 546.73 and 430.76 cm^−1^ indicated the stretching vibration of Si–O and Si–O–Si bending vibration [29]. The results confirm the effective incorporation of amino groups on the silica surface. In addition, XRD analysis of the nanoparticles was conducted at room temperature in the ranges of 10° to 90° in 2θ scale to determine whether the synthesized SiNPs were amorphous or crystalline. As shown in Figure 2d, the amorphous properties of the SiNPs were confirmed by a single broad diffraction peak in the XRD pattern at the 2θ value of 23.08° [30].

**Figure 3:**
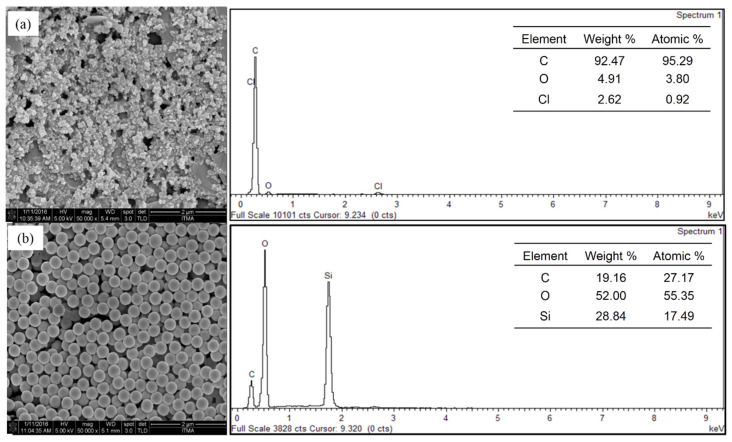
FESEM images coupled with EDX spectra for the (**a**) bare SPCE and (**b**) SiNPs/SPCE.

The surface morphology and elemental composition of the SiNPs/SPCE-modified electrode were characterized using FESEM and EDX, respectively. Figure 3a shows the distribution of carbon on the surface of the electrode which originates from the carbon-based SPCE. The Cl peak present in the EDX spectrum of the unmodified SPCE originates from the binder or solvent [31]. Meanwhile, the images illustrated in Figure 3b evidenced the distribution of silica on the electrode surface of the SPCE. As shown in the image, the presence of SiNPs on the electrode surface was confirmed and it was uniformly distributed on the working area of the screen-printed carbon electrode. The SiNPs exhibited a spherical shape and the size was in the range of 200–250 nm. The presence of silica and oxygen on the sparked electrode surface was confirmed by EDX analysis, as shown in Figure 3.

### 3.2. Electrochemical Characterization

**Figure 4:**
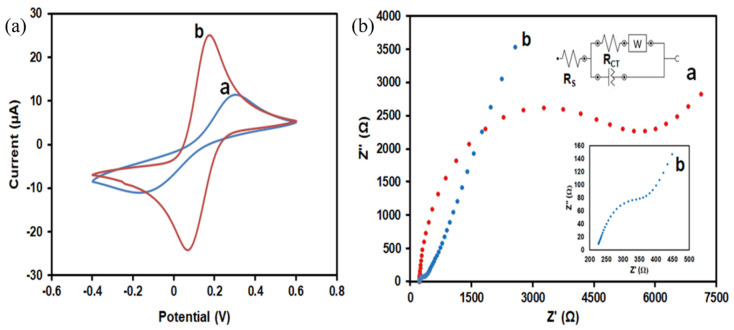
(**a**) Cyclic voltammetry (CV) responses of the a) bare SPCE and b) SiNPs/SPCE. (**b**) Nyquist plots of electrochemical impedance spectroscopy (EIS) for the a) bare SPCE and b) SiNPs/SPCE. The inset shows the Nyquist plot of the SiNPs/SPCE.

The electrochemical performance of the fabricated sensor was investigated using the CV technique in 5 mM of [Fe(CN)_6_]^3−/4−^ solution as a redox probe containing 0.1 M KCl as a supporting electrolyte at the potential range of −0.4 to 0.6 V, with a scan rate of 100 mV/s. As illustrated in Figure 4a, the unmodified electrode and SiNPs/SPCE exhibited a pair of well-defined redox due to the oxidation and reduction process of [Fe(CN)_6_]^3−/4−^. The SiNPs/SPCE produced a higher peak current compared with the bare SPCE. The anodic and cathodic peak current of the modified SPCE was significantly enhanced after the surface modification with SiNPs. It is probably due to the unique properties of SiNPs, which can increase the surface area and improve the electrode performance of the modified electrode. The peak separation was calculated for both the bare SPCE and SiNPs/SPCE. As can be seen from the result, the SiNPs/SPCE produces a small peak potential separation (0.17 V) compared with the bare SPCE (0.23 V), indicating faster kinetics of the electron transfer rate provided by the SiNPs.

The results were further confirmed by the electrochemical impedance spectroscopy (EIS) technique as shown in Figure 4b. EIS is an efficient method for probing the interfacial properties of surface-modified electrodes [32]. The measurements were recorded in 5 mM [Fe(CN)_6_]^3−/4−^ containing 0.1 M KCl at an electrode potential of 0.218 V, with a frequency range of 100 kHv to 0.01 Hz, and its equivalent Randle’s circuit. In the Nyquist plots of the EIS, the diameter of the semicircle equals the charge transfer resistance (Rct), which controls the electron transfer kinetics of the redox probe at the electrode surface. As shown in Figure 4b(a), the bare SPCE displays a large semicircle diameter with an Rct value of 12.9 kΩ at the high-frequency region. Meanwhile, the semicircle of the SiNPs/SPCE was significantly reduced with an Rct value of 121 Ω (Figure 4b(b)), demonstrating that the SiNPs-modified electrode surface formed a high electron conductor, which can be ascribed to the excellent conductivity of SiNPs. In summary, the EIS result shows the successful construction of the sensor.

**Figure 5:**
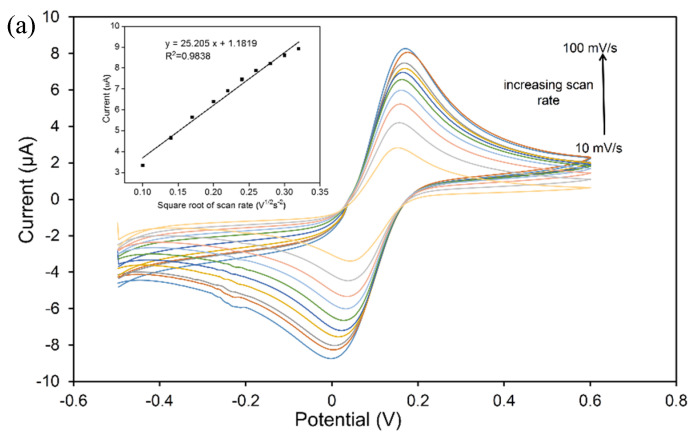

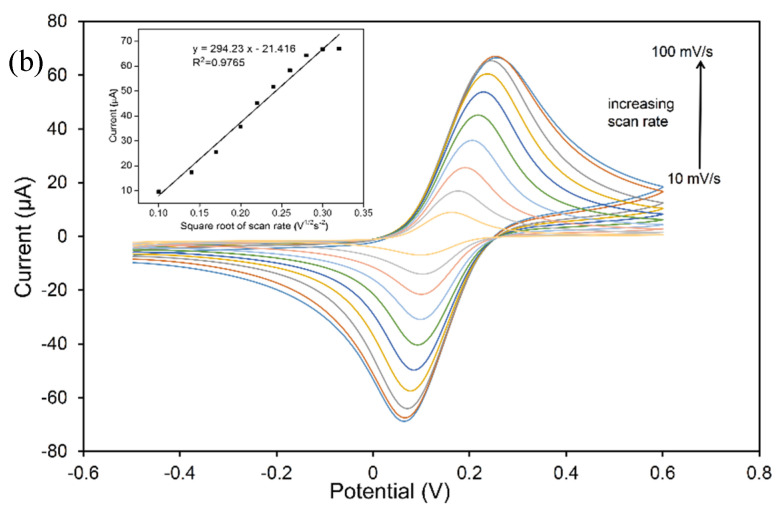
CV responses at different scan rates for the (**a**) bare SPCE and (**b**) SiNPs/SPCE. The insets show the corresponding linear calibration plot of the peak current against square root of the scan rate.

Meanwhile, the effective surface areas of the bare SPCE and SiNPs-modified SPCE were calculated using the Randles–Sevcik formula. The anodic peak current (Ipa) increased linearly with the square root of the scan rate. The effective surface area values of the bare SPCE and SiNPs/SPCE-modified electrode was calculated as 0.0309 and 0.4967 cm^2^, respectively. It was shown that the utilization of the SiNPs/SPCE as an electrochemical sensing material can enhance the effective surface area for about 16 times. Figure 5 shows that the oxidation peak current of the SiNPs/SPCE was proportional to the square root of the scan rate, which indicated that the electrochemical reaction of the SiNPs/SPCE behaved as a standard diffusion-controlled process [33]. It is worth noting that a direct response between the peak current and the bulk concentration was observed due to the interaction of the abundance active sites on the electrode surface with all incoming ion molecules, which is a crucial requirement for the electrochemical sensor [32]. This behavior makes the SiNPs/SPCE an effective electrode for the determination of As(III).

### 3.3. Parameters Optimization

**Figure 6:**
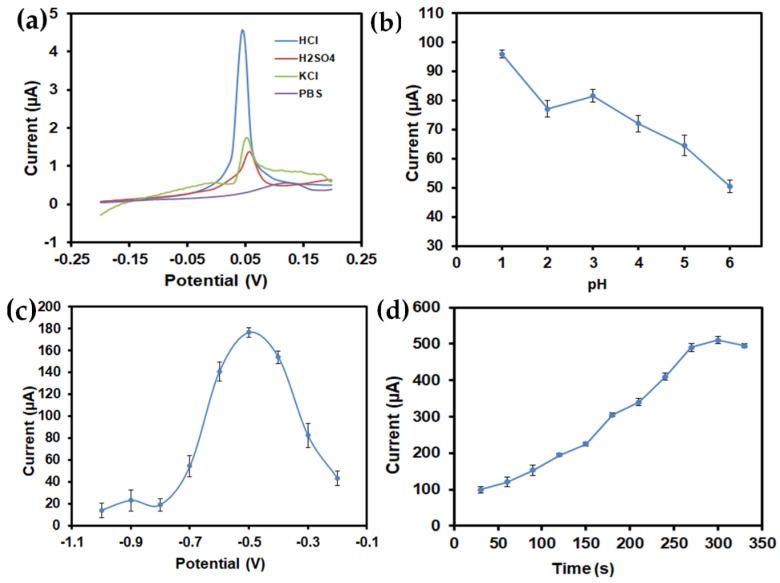
Effect of the (**a**) supporting electrolyte, (**b**) pH, (**c**) deposition potential, and (**d**) deposition time on the voltammetric response of the SiNPs/SPCE in the presence of 1 mg/L As(III).

The effect of experimental parameters such as supporting electrolytes, pH, deposition potential, and deposition time were examined in order to gain a high sensitivity of the electrochemical performance of the SiNPs/SPCE towards heavy metal ions, As(III). In the voltammetric analysis, the supporting electrolyte plays a vital role in increasing the conductivity of the analyte solution and maintaining the pH and ionic strength [21]. In this work, different kinds of supporting electrolytes were examined such as 1 M HCl, 1 M Tris-HCl, 1 M PBS, and 1 M KCl. Based on the analysis, 1 M HCl provides the optimum sensitivity and an intense peak current toward As(III) compared with other supporting electrolytes, as shown in Figure 6a. No visible peak current was observed in 1 M Tris-HCl, 1 M PBS, and 1 M KCl. Thus, 1 M HCl solution was selected as the optimized supporting electrolyte due to the sharp stripping peaks current observed.

The pH solution is another important factor that can affect the sensitive determination of As(III), since the speciation of arsenite varies with pH. As(III) exists predominantly in the low-pH region, which is less than 8 and gradually turns to anionic species beyond pH 8 [34]. A further phenomenon worth noting is the range of pH in which the surface charge densities of amine-functionalized SiNPs influence the confrontation of As(III). The nanoparticles retain a net positive charge at pH values below 6.84 and carry a net negative charge at a higher pH (pH > 6.84) [35,36]. It is, therefore, predicted that negatively charged arsenite ions could be effectively migrated to the electrode surface under acidic conditions. By considering both factors, the pH dependence studies were conducted by varying the pH values of HCl from 1 to 6 in response to the 10 µg/L of As(III). As shown in Figure 6b, it is clear that the highest peak current was observed at pH 1 and gradually decreased as the pH value increased. The trend was similar to that previously reported [23,32], showing that the reaction efficiency decreased at higher pH conditions, possibly due to the reduction of hydrogen that hindered the As(III) detection. Therefore, pH 1 was chosen as the optimum pH for all subsequent experiments.

The dependence of the anodic peak current concerning the applied deposition potential was analyzed from −1.0 to −0.2 V. As can be seen in Figure 6c, the intense stripping peak current appeared at −0.5 V during the detection of As(III). From the observation, the peak current increased significantly in the range of −0.1 to −0.5 V. However, as the deposition potential becomes more negative, the peak current leveled off due to the increasingly competitive H_2_ production which brought about the hydrogen evolution reaction occurring at the electrode surface at a more negative potential, which resulted in decreasing the current signals [37]. Hence, −0.5 V was adopted as the optimal deposition potential.

The influence of the deposition time on the current signals was investigated in the range of 30 to 330 s for As(III) detection. As illustrated in Figure 6d, the current peak increases with an increase in the deposition time from 30 to 300 s and decreases at 330 s after a prolonged accumulation time as a result of saturation. The decrease in the peak current was attributed to the limiting values of the deposited ions on the electrode surface at a higher deposition time [38]. Therefore, 300 s was chosen as the optimum deposition time and was used throughout this study.

### 3.4. Electrochemical Detection of As(III) on Modified Electrode

**Figure 7:**
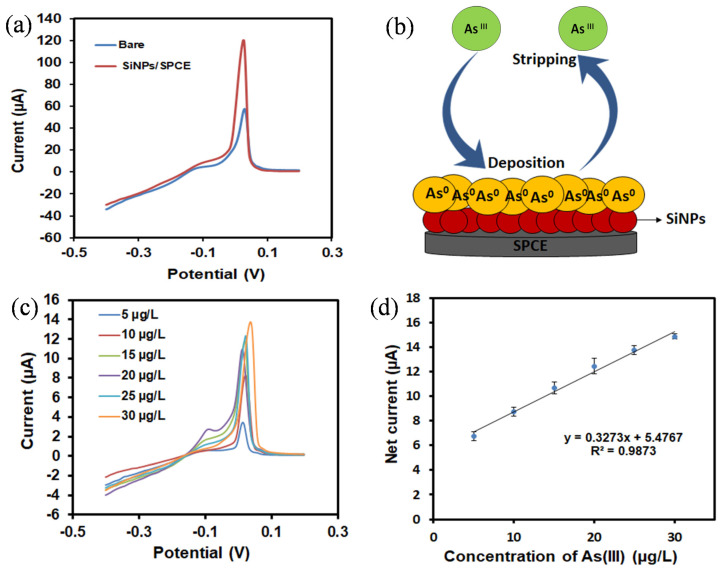
(**a**) Linear sweep anodic stripping voltammetry (LSASV) responses of the bare SPCE and SiNPs/SPCE in the presence of 1 mg/L As(III) in 1 M HCl solution (pH 1). (**b**) LSASV sensing mechanism of the SiNPs/SPCE towards As(III) detection. (**c**) LSASV responses of the SiNPs/SPCE towards As(III) in a concentration range of 5 to 30 µg/L and (**d**) the corresponding linear calibration plots of the net current against As(III) concentrations.

The applicability of the fabricated SiNPs-modified electrode towards As(III) detection was investigated using linear sweep anodic stripping voltammetry (LSASV). Figure 7a presents the electrochemical performance of the bare SPCE and SiNPs/SPCE, which was conducted in 1 mg/L of As(III) solution containing 1 M HCl (pH 1) as a supporting electrolyte at a deposition potential of 0.5 V and a deposition time of 300 s at the potential range of −0.4 to 0.2 V. As illustrated in Figure 7a, the bare SPCE displayed a weak oxidation peak and poor electrocatalytic activity compared with that by the SiNPs/SPCE-modified electrode. The higher sensitivity of the SiNPs/SPCE electrochemical sensors compared with the bare SPCE could be attributed to the distinctive electron transport and high adsorption properties of the SiNPs towards As(III). Thus, the fabricated SiNPs/SPCE showed better electrocatalytic activity, thereby capable of providing more remarkable electrochemical performances towards As(III) detection. Figure 7b demonstrates the sensing mechanism of arsenic detection using an APTES-functionalized SiNPs/SPCE. In this case, As(III) ions were adsorbed on the surface of the working electrode and reduced to As(0) by the employment of the applied deposition potential. This deposition process can be attributed towards the NH_2_-containing active groups on the SPCE and the conductivity of the SiNPs. Continually, the stripping mechanism was further performed by supplying a specific stripping voltage, which caused the reoxidation of As(0) to As(III) ions and their removal from the electrode surface [39]. The corresponding oxidation current (stripping current) is measured as a function of the scan potential, allowing the highly selective and sensitive detection of As(III) ions via the SiNPs/SPCE sensor through the LSASV approach.

Under the optimized experimental conditions, the performance of the SiNPs/SPCE sensor was further analyzed using an LSASV measurement in the presence of various concentrations of As(III). Figure 7c shows the LSASV responses of the SiNPs/SPCE for a range of As(III) concentrations from 5 to 30 µg/L. It was found that the stripping current had increased when the value of the arsenite concentration was increased. This behavior can be accredited to the presence of a moiety that actively reacted to the positively charged target analyte [23], indicating that the peak response was controlled by the amount of As(III) ions adsorbed on the surface of the electrode modifiers, SiNPs. The calibration plot was linear over the concentration range of 5 to 30 µg/L of As(III), with the regression equation of IP (µA) = 1.6449C (µg/L) + 5.1673 (R^2^ = 0.9933), as depicted in Figure 7d. The findings showed that the detection of As(III) in aqueous media had been efficiently obtained, even at a very low concentration of arsenic. The LOD was further determined using the equation of LOD = 3S_b_/b, where S_b_ is the standard deviation of the blank measurements (n = 3), and b is the slope of the calibration curve [40]. The LOD was calculated as 6.2 µg/L, which is lower than the maximum guide value of 10 µg/L by the World Health Organization (WHO). The LOD obtained in the present study was comparable to those reported previously, as in Table 1. Although previous reports had achieved higher sensitivity and lower detection limits, some issues still arise, particularly in terms of its expensiveness, complicated fabrication protocol, and the use of toxic materials. It is imperative to note that in addition to offering a competitive limit of detection in comparison with existing reports, critically, the SiNPs/SPCE also allows for the detection of As(III) at a lower LOD than that observed using IrO_2_/GCE [41] and TiO_2_/GSE [42], with the linear range being wider than reported using AuNPs/SPE [18] and Pt/GCE [43].

The reproducibility, repeatability, and stability of the developed sensor were investigated under optimum conditions and the results are presented in Table 2. The reproducibility was examined using a series of six sets of a similarly constructed SiNPs/SPCE. The relative standard deviation responses (RSD) were found to be 4.78%, showing good reproducibility. Meanwhile, the repeatability of the fabricated sensor also was evaluated for six repetitive measurements of a single SiNPs/SPCE in 10 µg/L As(III) solution. The RSD value of 12.48% suggests that the fabricated sensors are not repeatable, and hence allows for a single-shot (disposable) analysis. Additionally, the stability of the SiNPs/SPCE also was studied by recording the current each day using a single SiNPs/SPCE electrode. The modified electrode was stored at room temperature. From the results obtained, the responses current decreases from day-to-day. This shows that the best current responses can only be achieved when a freshly prepared SiNPs/SPCE is used. From the observation, the color of the reference site turned black after a few days at room temperature due to the oxidation process.

### 3.5. Interference Measurements

**Figure 8:**
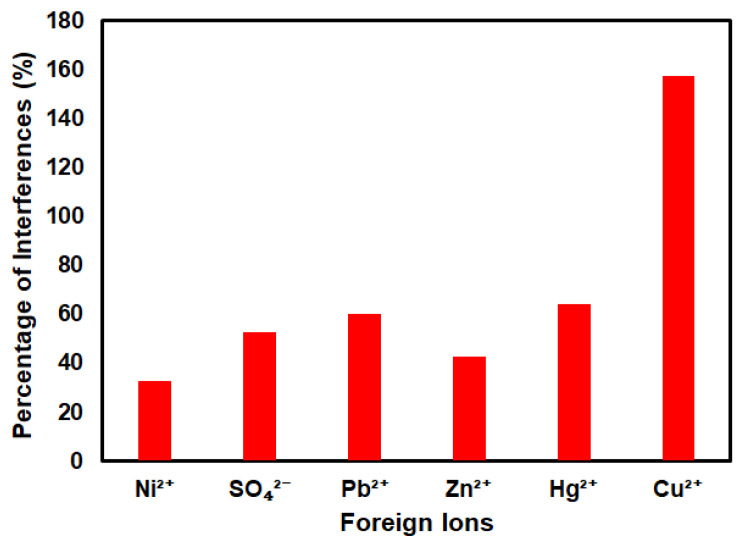
Interference study of the SiNPs/SPCE in the presence of various competitive foreign ions.

Studies of a possible interference from foreign ions that exist in natural water have been conducted. Figure 8 shows the effect of various foreign ions such as Hg^2+^, SO_4_^2−^, Pb^2+^, Zn^2+^, Ni^2+^, and Cu^2+^, which were tested for the interference study of the SiNPs/SPCE-modified electrode under optimum conditions. The degree of interference was studied by adding some possible interfering metals into 1 M HCl containing 10 µg/L As(III) at 1:1 of the As(III) concentration to foreign substances. The results show that the fabricated sensor has good selectivity towards As(III) ions. Only Cu(II) ions were found to suppress the peak current. It is due to the natural existence of Cu(II) in the water system, and it was found present in relatively high levels in the world’s water supply. During the deposition step in the electrochemical measurement, copper and arsenic were co-deposited on the electrode and formed an intermetallic compound Cu_3_As_2_ alloy [49]. Thus, due to this reason, Cu(II) shows a significant interference in arsenic detection. This finding is in good agreement with previous studies that reported a major interference from Cu(II) ions in the electrochemical detection of As(III) in water [49].

### 3.6. Real Sample Analysis

A series of tests was performed on real water samples to evaluate the practical application of the SiNPs/SPCE towards detecting the target analyte, As(III), in the environment. The samples were collected from river water (Balok River, Kuantan, Malaysia) and tap water (Universiti Putra Malaysia). Prior to the electrochemical analysis, the water samples were diluted with 0.1 M HCl, and no further treatment was performed. The unknown concentrations of As(III) were initially tested. Then, 10 mg/L of As(III) was spiked into the samples and measured using the developed sensor. It is clear that no apparent peak of As(III) was observed, suggesting that no As(III) was found in the diluted samples for Tap water A and River water A, respectively. However, when As(III) was spiked into the samples, an obvious signal change was detected corresponding to the As(III) concentration increasing in the samples of Tap water B and River water B, respectively. The results obtained by the proposed method were comparable to those obtained by the inductively coupled plasma-mass spectrometry (ICP-MS) analysis as presented in Table 3. The recovery test was further studied to validate the accuracy of the developed SiNPs/SPCE sensor for arsenic detection by spiking 10 mg/L of As(III). As there was a 97.6% recovery for the Tap water B sample and 98.5% recovery for the River water B sample, these findings demonstrate that the proposed sensor is feasible and has a good applicability for real samples application.

## 4. Conclusions

An efficient electrochemical sensor for As(III) detection with high electrochemical performance was successfully developed using the LSASV technique. The fabricated sensor exhibited good electrical performance and provided an excellent response towards As(III) detection with high selectivity and sensitivity. A reasonable limit of detection was obtained from the calibration study, in which the obtained LOD value was 6.2 µg/L, which was below the threshold limits of drinking water, as suggested by WHO, 10 µg/L. The SiNPs/SPCE-modified sensor also showed good reproducibility with an RSD value of 4.78 %. Combined with the disposable SPCE, this cost-effective analytical assay has proven to be successful for the detection of As(III) in real water samples and continues to hold great promise to be used as a screening platform for environmental analysis.

## Figures and Tables

**Table 1:** Comparative study on the performance of different electrochemical sensors for arsenite detection.

Modified Electrode	Detection Method	Linear Range (µg/L)/ppb	LOD (µg/L)/ppb	Ref.
AuNPs/SPE	CV	0.5–12	0.22	[18]
IrO_2_/GCE	DPV	0–80	7.7	[41]
TiO_2_/GSE	LSV	10–80	10	[42]
Pt/GCE	CV/LSV	2–14	2.1	[43]
Au-Cu/GCE	CV	30–130	5.64	[44]
Au- MnFe_2_O_4_/GCE	SWASV	0–60	3.37	[45]
EG-Bi/GCE	SWASV	20–100	5.0	[46]
MnFe_2_O_4_/GE	SWASV	0–100	1.95	[47]
SnO_2_/GE	SWASV	5–300	5.0	[48]
SiNPs/SPCE	LSASV	5–30	6.2	This work

AuNPs: gold nanoparticles; Au-Cu: gold-copper; Au-MnFe_2_O_4_: gold-manganese ferrite; EG-Bi: exfoliated graphite-bismuth; IrO_2_: iridium oxide; MnFe_2_O_4_: manganese ferrite; Pt: platinum; SnO_2_: tin dioxide; TiO_2_: titanium dioxide; SiNPs: silica nanoparticles; GE: gold electrode; GCE: glassy carbon electrode; GSE: gold strip electrode; SPE: screen-printed electrode; SPCE: screen-printed carbon electrode; CV: cyclic voltammetry; DPV: differential pulse voltammetry; LSV: linear sweep voltammetry; LSASV: linear swept anodic stripping voltammetry; SWASV: square wave anodic stripping voltammetry.

**Table 2:** Reproducibility, repeatibility, and stability study of the SiNPs/SPCE sensor.

No. of Sample/Day	Reproducibility (Peak Current, µA)	Repeatability (Peak Current, µA)	Stability (Peak Current, µA)
1	56.75	52.47	55.32
2	51.00	52.08	52.83
3	50.65	48.93	50.04
4	53.65	45.99	47.87
5	54.65	37.99	44.34
Mean	53.34	47.49	50.08
Standard deviation	2.55	5.93	4.26
RSD (%)	4.78	12.48	8.51

**Table 3:** Real sample analysis using the developed SiNPs/SPCE sensor for As(III) detection.

Sample	As (III) Spiked (mg/L)	SiNPs/SPCE		ICP-MS
		As (III) Found (mg/L)	Recovery (%)	As (III) Found (mg/L)
Tap water A	0	0.68 ± 0.01	–	1.09 ± 0.26
Tap water B	10	10.54 ± 0.43	97.6	10.37 ± 0.62
River water A	0	0.95 ± 0.18	–	0.58 ± 0.01
River water B	10	10.77 ± 0.65	98.5	10.84 ± 0.79

## References

[1] Karthika A., Selvarajan S., Karuppasamy P., Suganthi A., Rajarajan M. (2019). A novel highly efficient and accurate electrochemical detection of poisonous inorganic Arsenic(III) ions in water and human blood serum samples based on SrTiO3/β-cyclodextrin composite. J. Phys. Chem. Solids.

[2] Wang W., Bao N., Yuan W., Si N., Bai H., Li H., Zhang Q. (2019). Simultaneous determination of lead, arsenic, and mercury in cosmetics using a plastic based disposable electrochemical sensor. Microchem. J..

[3] Luong J.H.T., Lam E., Male K.B. (2014). Recent advances in electrochemical detection of arsenic in drinking and ground waters. Anal. Methods.

[4] Yuan Y., Zhu X., Wen S., Liang R., Zhang L., Qiu J. (2018). Electrochemical assay for As(III) by combination of highly thiol-rich trithiocyanuric acid and conductive reduced graphene oxide nanocomposites. J. Electroanal. Chem..

[5] Bu L., Liu J., Xie Q., Yao S. (2015). Anodic stripping voltammetric analysis of trace arsenic(III) enhanced by mild hydrogen-evolution at a bimetallic Au-Pt nanoparticle modified glassy carbon electrode. Electrochem. Commun..

[6] Vega L., Styblo M., Patterson R., Cullen W., Wang C., Germolec D. (2001). Differential effects of trivalent and pentavalent arsenicals on cell proliferation and cytokine secretion in normal human epidermal keratinocytes. Sect. Title Toxicol..

[7] Domínguez-álvarez J. (2020). Talanta Capillary electrophoresis coupled to electrospray mass spectrometry for the determination of organic and inorganic arsenic compounds in water samples. Talanta.

[8] Dilbaghi N., Kumar S., Kim K.-H., Chaudhary G.R., Bhanjana G., Mehta N. (2018). Novel electrochemical sensing of arsenic ions using a simple graphite pencil electrode modified with tin oxide nanoneedles. J. Mol. Liq..

[9] Kumar P., Devi P., Jain R., Saini A., Noetzel R. (2019). Electrochemical Detection of Trace Arsenic(III) by functionalized In0.38Ga0.62N/Si(1 1 1) electrode. Mater. Lett..

[10] Núñez C., Arancibia V., Gómez M. (2016). Determination of arsenic in the presence of copper by adsorptive stripping voltammetry using pyrrolidinedithiocarbamate or diethyl dithiophosphate as chelating-adsorbent agents. Effect of CPB on the sensitivity of the method. Microchem. J..

[11] Saha S., Sarkar P. (2016). Talanta Differential pulse anodic stripping voltammetry for detection of As(III) by Chitosan-Fe(OH) 3 modi fi ed glassy carbon electrode: A new approach towards speciation of arsenic. Talanta.

[12] Gu H., Yang Y., Chen F., Liu T., Jin J., Pan Y., Miao P. (2018). Electrochemical detection of arsenic contamination based on hybridization chain reaction and RecJfexonuclease-mediated amplification. Chem. Eng. J..

[13] Murugappan K., Lee J., Silvester D.S. (2011). Comparative study of screen printed electrodes for ammonia gas sensing in ionic liquids. Electrochem. Commun..

[14] Kolliopoulos A., Metters J.P., Banks C. (2013). Screen printed graphite electrochemical sensors for the voltammetric determination of antimony(III). Anal. Methods.

[15] Metters J.P., Kadara R.O., Banks C.E. (2011). New directions in screen printed electroanalytical sensors: An overview of recent developments. Analyst.

[16] Couto R.A.S., Lima J.L.F.C., Quinaz M.B. (2016). Recent developments, characteristics and potential applications of screen-printed electrodes in pharmaceutical and biological analysis. Talanta.

[17] Pan F., Chen D., Zhuang X., Wu X., Luan F., Zhang S., Wei J., Xia S., Li X. (2018). Fabrication of gold nanoparticles/l-cysteine functionalized graphene oxide nanocomposites and application for nitrite detection. J. Alloy. Compd..

[18] Trachioti M.G., Karantzalis A.E., Hrbac J., Prodromidis M.I. (2019). Low-cost screen-printed sensors on-demand: Instantly prepared sparked gold nanoparticles from eutectic Au / Si alloy for the determination of arsenic at the sub-ppb level. Sens. Actuators B Chem..

[19] Lan Y., Luo H., Ren X., Wang Y., Liu Y. (2012). Anodic stripping voltammetric determination of arsenic(III) using a glassy carbon electrode modified with gold-palladium bimetallic nanoparticles. Microchim. Act..

[20] Boruah B.S., Daimari N.K., Biswas R. (2019). Functionalized silver nanoparticles as an effective medium towards trace determination of arsenic(III) in aqueous solution. Result. Phys..

[21] Li J., Chen L., Lou T., Wang Y. (2011). Highly Sensitive SERS Detection of As^3+^ Ions in Aqueous Media using Glutathione Functionalized Silver Nanoparticles. ACS Appl. Mater. Interfaces.

[22] Ismail S., Yusof N.A., Abdullah J., Rahman S.F.A. (2020). Development of Electrochemical Sensor Based on Silica/Gold Nanoparticles Modified Electrode for Detection of Arsenite. IEEE Sens. J..

[23] Kaur R., Rana S., Singh R., Kaur V., Narula P. (2019). A Schiff base modified graphene oxide film for anodic stripping voltammetric determination of arsenite. Microchim. Acta.

[24] Chimezie A.B., Hajian R., Yusof N.A., Woi P.M., Shams N. (2017). Fabrication of reduced graphene oxide-magnetic nanocomposite (rGO-Fe3O4) as an electrochemical sensor for trace determination of As(III) in water resources. J. Electroanal. Chem..

[25] Mathelié-guinlet M., Cohen-Bouhacina T., Gammoudi I., Martin A., Béven L., Delville M.H., Grauby-Heywang C. (2019). Silica nanoparticles-assisted electrochemical biosensor for the rapid, sensitive and speci fi c detection of Escherichia coli. Sens. Actuators B Chem..

[26] Pérez-ràfols C., Serrano N., Díaz-cruz J.M., Ariño C., Esteban M. (2016). Glutathione modi fi ed screen-printed carbon nano fi ber electrode for the voltammetric determination of metal ions in natural samples. Talanta.

[27] Reddy S., Oldham D., Fini E.H., Zhang L. (2019). Surface functionalization of silica nanoparticles to enhance aging resistance of asphalt binder. Constr. Build. Mater..

[28] Sekar S., Kaur N., Lee S., Kim D. (2018). Rapid Sonochemical Synthesis of Spherical Silica Nanoparticles Derived from Brown Rice Husk. Ceram. Int..

[29] Ye X., Hao C., Yang J., Sun R. (2018). Biointerfaces In fluence of modi fi ed silica nanoparticles on phase behavior and structure properties of DPPC monolayers. Colloids Surf. B.

[30] Verma J., Bhattacharya A. (2018). Analysis on Synthesis of Silica Nanoparticles and its Effect on Growth of T. Harzianum & Rhizoctonia Species. Biomed. J. Sci. Tech. Res..

[31] Sunon P., Wongkaew P., Johns J., Johns N. (2018). Characterization of Cerium Oxide-Chitosan Nanocomposite—Modified Screen Printed Carbon Electrode and Application in Melatonin Determination. Int. J. GEOMATE.

[32] Chen D., Zhuang X., Zhai J., Zheng Y., Lu H., Chen L. (2018). Preparation of highly sensitive Pt nanoparticles-carbon quantum dots/ionic liquid functionalized graphene oxide nanocomposites and application for H_2_O_2_ detection. Sens. Actuators B Chem..

[33] Manzanares A., Murtom L. (2019). Thermodiffusion of sodium polystyrene sulfonate in a supporting electrolyte. Electrochim. Acta.

[34] Lee S.M., Lalhmunsiama T., Tiwari D. (2015). Porous hybrid materials in the remediation of water contaminated with As(III) and As(V.). Chem. Eng. J..

[35] Zienkiewicz-Strzałka M., Deryło-Marczewska A., Kozakevych R.B. (2018). Silica nanocomposites based on silver nanoparticles-functionalization and pH effect. Appl. Nanosci..

[36] Lalmalsawmi J., Zirlianngura D., Tiwari S., Lee M. (2020). Low cost, highly sensitive and selective electrochemical detection of arsenic(III) using silane grafted based nanocomposite. Environ. Eng. Res..

[37] Fakude C.T., Arotiba O.A., Moutloali R., Mabuba N. (2019). Nitrogen-doped Graphene Electrochemical Sensor for Selenium(IV) in Water. Int. J. Electrochem. Sci..

[38] Wei Y., Yang R., Liu J.H., Huang X.J. (2013). Selective detection toward Hg(II) and Pb(II) using polypyrrole/carbonaceous nanospheres modified screen-printed electrode. Electrochim. Acta.

[39] Durai L., Badhulika S. (2020). Ultra-selective, trace level detection of As3+ ions in blood samples using PANI coated BiVO4 modified SPCE via differential pulse anode stripping voltammetry. Mater. Sci. Eng. C.

[40] Aina F., Manan A., Weng W., Abdullah J., Azah N., Ahmad I. (2019). Materials Science & Engineering C Nanocrystalline cellulose decorated quantum dots based tyrosinase biosensor for phenol determination. Mater. Sci. Eng. C.

[41] Mafakheri E., Salimi A., Hallaj R., Ramazani A., Kashi M.A. (2011). Synthesis of Iridium Oxide Nanotubes by Electrodeposition into Polycarbonate Template: Fabrication of Chromium(III) and Arsenic(III) Electrochemical Sensor. Electroanalysis.

[42] Zhang X., Zeng T., Hu C., Hu S. (2016). Studies on fabrication and application of arsenic electrochemical sensors based on titanium dioxide nanoparticle modified gold strip electrodes. Anal. Methods.

[43] Dai X., Compton R.G. (2006). Detection of As(III) via oxidation to As(v) using platinum nanoparticle modified glassy carbon electrodes: Arsenic detection without interference from copper. Analyst.

[44] Yang M., Guo Z., Li L.N., Huang Y.Y., Liu J.H., Zhou Q., Chen X., Huang X.J. (2016). Electrochemical determination of arsenic(III) with ultra-high anti-interference performance using Au–Cu bimetallic nanoparticles. Sens. Actuators B Chem..

[45] Zhou S., Han X., Fan H., Liu Y. (2016). Electrochemical Sensing toward Trace As(III) Based on Mesoporous MnFe_2_O_4_/Au Hybrid Nanospheres Modified Glass Carbon Electrode. Sensors.

[46] Ndlovu T., Mamba B.B., Sampath S., Krause R.W., Arotiba O.A. (2014). Voltammetric detection of arsenic on a bismuth modified exfoliated graphite electrode. Electrochim. Acta.

[47] Zhou S.F., Han X.J., Fan H.L., Zhang Q.X., Liu Y.Q. (2015). Electrochemical detection of As(III) through mesoporous MnFe_2_O_4_ nanocrystal clusters by square wave stripping voltammetry. Electrochim. Acta.

[48] Jiang T.J., Guo Z., Liu J.H., Huang X.J. (2016). Gold electrode modified with ultrathin SnO_2_ nanosheets with high reactive exposed surface for electrochemical sensing of As(III). Electrochim. Acta.

[49] Idris A.O., Mafa J.P., Mabuba N., Arotiba O.A. (2016). Dealing with interference challenge in the electrochemical detection of As(III)—A complexometric masking approach. Electrochem. Commun..

